# CSAMT-Driven Feasibility Assessment of Beishan Underground Research Laboratory

**DOI:** 10.3390/s25144282

**Published:** 2025-07-09

**Authors:** Zhiguo An, Qingyun Di, Changmin Fu, Zhongxing Wang

**Affiliations:** Key Laboratory of Deep Petroleum Intelligent Exploration and Development, Institute of Geology and Geophysics, Chinese Academy of Sciences, Beijing 100029, China; qydi@mail.iggcas.ac.cn (Q.D.); fcm168@mail.iggcas.ac.cn (C.F.); zxwang@mail.iggcas.ac.cn (Z.W.)

**Keywords:** Beishan, Xinchang site, underground research laboratory (URL), CSAMT exploration

## Abstract

The safe disposal of high-level radioactive waste (HLW) is imperative for sustaining China’s rapidly expanding nuclear power sector, with deep geological repositories requiring rigorous site evaluation via underground research laboratories (URLs). This study presents a controlled-source audio-frequency magnetotellurics (CSAMT) survey at the Xinchang site in China’s Beishan area, a region dominated by high-resistivity metamorphic rocks. To overcome electrical data acquisition challenges in such resistive terrains, salt-saturated water was applied to transmitting and receiving electrodes to enhance grounding efficiency. Using excitation frequencies of 9600 Hz to 1 Hz, the survey achieved a 1000 m investigation depth. Data processing incorporated static effect removal via low-pass filtering and smoothness-constrained 2D inversion. The results showed strong consistency between observed and modeled data, validating inversion reliability. Borehole correlations identified a 600-m-thick intact rock mass, confirming favorable geological conditions for URL construction. The study demonstrates CSAMT’s efficacy in characterizing HLW repository sites in high-resistivity environments, providing critical geophysical insights for China’s HLW disposal program. These findings advance site evaluation methodologies for deep geological repositories, though integrated multidisciplinary assessments remain essential for comprehensive site validation. This work underscores the feasibility of the Xinchang site while establishing a technical framework that is applicable to analogous challenging terrains globally.

## 1. Introduction

The geological disposal of high-level radioactive waste (HLW) represents a critical challenge for nations utilizing nuclear energy. Internationally, deep geological repositories at depths of 500–1000 m are considered the most viable solution, leveraging natural geological barriers for long-term radionuclide isolation. Previous electromagnetic investigations at established sites include studies at the Waste Isolation Pilot Plant in Carlsbad, USA [1]; Yucca Mountain, USA [2]; and the Canadian Underground Research Laboratory [3]. In China’s pre-selected Beishan area—an arid region in northwestern Gansu Province with an annual precipitation below 80 mm and Proterozoic metamorphic granite bedrock [4]—we conducted a controlled-source audio-frequency magnetotellurics (CSAMT) survey using a Phoenix Geophysics V8 system in 2009 in order to investigate the spatial distribution, structural characteristics, and integrity of rock masses within the 500–1000 m depth range.

For the subsurface characterization of deep geological repositories (>500 m burial depth), electromagnetic investigation systems require operational depths exceeding 1 km. The CSAMT method has demonstrated particular efficacy in such deep-probing applications. As an active electromagnetic surveying technique employing artificial signal sources (typically grounded dipoles or horizontal loop transmitters), CSAMT overcomes the critical limitations of passive magnetotelluric methods through controlled signal generation and repeatable transmission protocols [5].

This frequency-domain electromagnetic approach has proven effective across diverse geophysical investigations including, but not limited to, mineral prospecting, geothermal reservoir mapping, hydrocarbon exploration, and hydrogeological studies [6,7,8]. Its technical advantages are particularly evident in the following two operational scenarios: (1) urbanized or industrially active areas where cultural electromagnetic interference obscures natural telluric signals and (2) survey environments requiring enhanced signal-to-noise ratios at audio-frequencies (0.1–10 kHz) where ambient electromagnetic fields exhibit suboptimal amplitudes [9,10].

What are the morphology, scale, and boundary characteristics of the Xinchang rock mass at depth? This study aims to clarify whether it possesses continuous and stable spatial occurrence conditions. Additionally, we aim to characterize the occurrence features of fractures and crushed zones, as well as assessing whether they pose a potential threat to waste isolation performance. Given that the Xinchang site is surrounded by two nearly east–west-trending faults and two northeast-trending faults, five profiles oriented at NE30° were deployed to reveal whether underground northwest-trending geological structures or adverse geological features exist. The three objectives utilized in this study are as follows: (1) mapping fault systems to 1000 m depth, (2) identifying hidden fracture zones through resistivity contrasts, and (3) correlating subsurface structures with hydrogeological features.

To overcome the challenge of high contact resistance caused by the direct placement of electrodes on exposed bedrock, an innovative solution employing non-woven fabric impregnated with salt-saturated water was implemented. This significantly enhanced electrode grounding effectiveness, ensuring feasible data acquisition.

The CSAMT exploration in Beishan has revealed the deep structural stability and fracture risks of the Xinchang rock mass, providing critical geophysical validation for the safe site selection of HLW disposal repositories.

## 2. Lithologies in the Study Area

The lithostratigraphic framework of the study area demonstrates a metamorphic-dominated terrain, with sporadic quaternary deposits (<5% areal coverage) unconformably overlying Precambrian basement rocks. The orthometamorphic series, constituting ~85% of exposures, comprises the following two principal units [11]:

(1) Mesoproterozoic Gneissic Granodiorite (Pt_2_^2^H)
  - Medium- to fine-grained texture with augen gneiss fabric;
  - Predominantly exposed in the central sector;
  - Interpreted as anatectic products of ancient continental crust.
(2) Caledonian Metamorphic Monzogranite (O_1_X)
  - Exhibits porphyroblastic textures with K-feldspar megacrysts;
  - Extensively distributed across northern and southwestern domains;
  - Notably forms continuous outcrops along northeastern survey transects.

**Figure 1:**
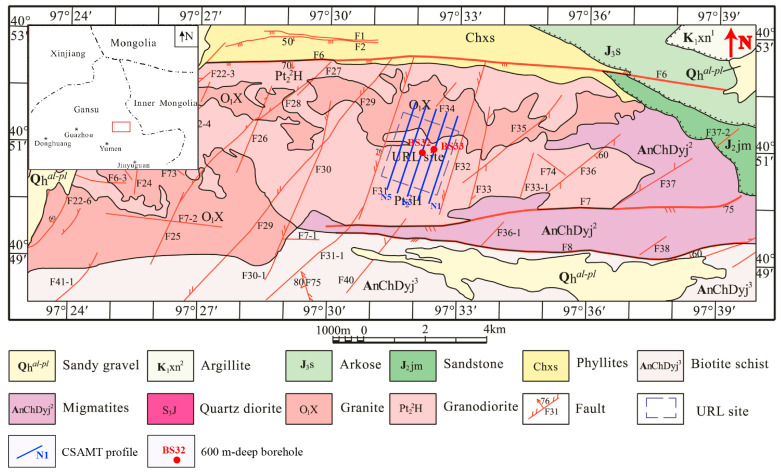
Geological map of the Xinchang site (modified according to An et al., 2013 [12] and Wang et al., 2018 [4]). The orientation of the survey profiles was designed to incorporate both the dominant structural strike and recommendations from the Beijing Research Institute of Uranium Geology. Thick lines (e.g., F6, F7, and F8) denote primary structures, while thin lines represent secondary faults.

Parametamorphic lithologies (15% exposure), restricted to eastern and southern sectors (Figure 1), include the following:

(3) Paleoproterozoic–Archean Migmatites (AnChDyj2)
  - Stromatic to nebulitic structures;
  - Syn-tectonic garnet-bearing assemblages.
(4) Pre-Changcheng Schist Series (AnChDyj3).
  - Dominated by biotite–quartz schists with subordinate marble interbeds;
  - Preserves relict marine clastic–carbonate protoliths.

Petrogenetic analysis reveals these metamorphic units underwent greenschist to amphibolite facies conditions (T = 400–550 °C, P = 4–6 kbar), consistent with polyphase orogenic events during the Columbia to Rodinia supercontinent cycles.

## 3. Structure in the Study Area

The Xinchang site is located within the central segment of the Liuyuan–Tiancang fold belt in the Beishan sector of the Tianshan–Yinshan Latitudinal Tectonic System. It belongs to the Erdaojing–Xiqianluzi–Jiusidunbei fault fold belt. This domain exhibits well-developed fold structures and fault systems, with dominant compressional and transpressional fault zones and fold axial planes trending in the EW to sub-EW orientations. Neotectonic activity manifests as episodic crustal uplift–subsidence cycles, which have shaped the present-day geological architecture and geomorphic morphology [13].

Based on mechanical properties, the faults can be categorized into two types.

### 3.1. Compressional and Transpressional Faults

These faults predominantly strike the EW to sub-EW directions, parallel to the regional strata trend. As dominant structures controlling tectonic framework, they truncate Mesozoic and older strata/intrusions, demonstrating polyphase reactivation. Most faults extend 10–20 km in length with widths of tens of meters, often forming valleys in modern topography. Key examples include the Jijing–Hongliujingnanshan Fault (F6), the Yuanyangou Fault (F7), and the Yuanyangounan Fault (F8) (Figure 1). Notably, some faults (e.g., F6) exhibit a composite brittle–ductile deformation. The F6 fault represents the reactivation of an ancient regional-scale ductile shear zone, evidenced by intensively developed stretching lineations from early deformation phase, and a 500–1000-m-wide belt of augen migmatites within the shear zone.

### 3.2. Strike–Slip and Transtensional Faults

These faults are secondary structures to the compressional/transpressional systems. Typically extending 2–5 km in length, most strike NNE to NE (minor NW trends) with dips of 30–50°, exhibiting dominant sinistral (left-lateral) motion. Fault zones commonly contain silica-cemented breccias and locally fault gouge. Displacements of strata/dykes range from meters to tens of meters across fault planes. Representative examples include the Xinchang Fault (F28), the Xinchangdong Fault (F26), the Jijing Fault (F31), and the Jijingdong Fault (F32). These faults compartmentalize the granite massif into discrete fault-bounded blocks. Fault F31, with a length of 5.5 km, strikes NNE and dips 70–83°, exhibiting sinistral transtensional strike–slip characteristics. It has a width of 1.0–2.6 m and is composed of single or multiple subparallel fault planes, forming a fault breccia zone. The fault rocks consist of silicified breccias, with host rocks exhibiting silicification alteration, and early-stage lamprophyre veins intruding into the structure. The F32 fault is a sinistral transtensional fault with a total length of 6.1 km, striking NE and dipping at 60–80°. It exhibits a width of 0.3–3.1 m and is composed of single or multiple subparallel fault planes. The fault zone contains silicified breccia, cataclasite, mylonite, and brecciated monzogranite as tectonic rocks. Early-stage monzogranite dikes and lamprophyre dikes are emplaced along the fault. Fracture F34 is a tensile fault with a length of 1.3 km, striking NE. It dips at 72–86° and has a width of 0.2–0.9 m. The tectonic rocks within the fracture zone are predominantly silicified breccia, with minor occurrences of cataclasite [14].

The Xinchang area exhibits relatively weak neotectonic intensity, primarily manifesting as differential uplift–subsidence. Since the Eocene (Paleogene), three-tiered planation surfaces have developed, but the absence of tertiary deposits indicates the following: stable weathering–denudation, uplift, and renewed denudation stability. This multistage uplift generated a prominent EW-trending arcuate structure—the Xinchang–Hongliujingnanshan uplift belt. This feature extends ~20 km (EW) with a 4–7 km (NS) width, elevated 100–150 m above adjacent terrains.

## 4. CSAMT Data Acquisition and Processing

### 4.1. CSAMT Raw Data

**Figure 2:**
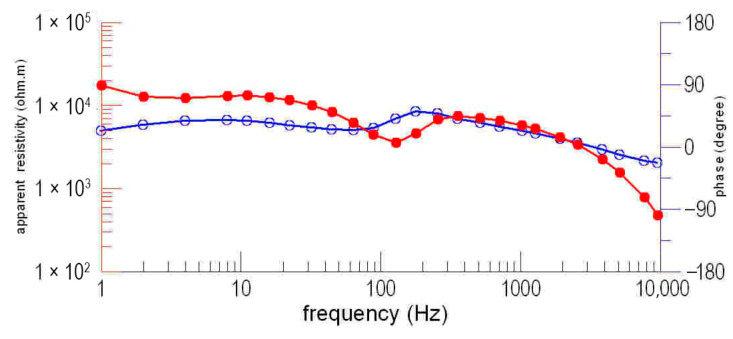
CSAMT sounding curve in the study area. The red dots and lines represent apparent resistivity, while blue dots and lines denote the impedance phase.

The electromagnetic survey employed a Phoenix TXU-30 transmitter positioned 12 km southeast of Profile N1 in a dipole-parallel configuration, delivering a 9.5 A current through a 1.4 km transmitter dipole. To overcome high-contact resistance in arid metamorphic granite terrain (*ρ* > 5000 Ω), a pre-acquisition protocol was implemented, as follows: (1) Pb-PbCl_2_ non-polarizable electrodes; (2) daily saline irrigation (26.4% *w*/*w* saturated brine) at electrode sites; and (3) layered interface optimization using 50 × 50 cm saline-saturated geotextile beneath bentonite–clay mixtures. CSAMT data acquisition utilized a 9-channel V8 system, recording 25 frequencies (9600–1 Hz). Profiles N1, N2, N4, and N5 had a point spacing of 40 m, with 77 sounding points per profile, while profile N3 employed a 20 m point spacing with 154 sounding points. The purpose of selecting this frequency band is to ensure both sufficient detection depth and adequate resolution. The signal quality was enhanced through adaptive stacking, achieving signal-to-noise ratios exceeding 60 dB. Data processing applied Hanning-windowed static shift correction and 2D Occam inversion with L2-norm regularization (RMS < 3%), yielding Cagniard apparent resistivity and impedance phase estimates. The smooth sounding curve with distortion-free frequencies indicates high-quality data (Figure 2).

Figure 2 presents the apparent resistivity and phase response profiles observed above intact rock formations. At high-frequency regimes, the apparent resistivity exhibits a baseline value of approximately 1000 Ω·m. With decreasing frequency, the apparent resistivity demonstrates a gradual upward trend, reaching a maximum of 10,000 Ω·m. This progression transitions through an intermediate region characterized by minimal variations in resistivity. On the sounding curve within the 160–60 Hz segment, it manifests as a transition. At approximately 60 Hz, the resistivity continues to escalate, reflecting the dominance of skin effect phenomena in the electromagnetic response of the intact rock medium. Derived from skin depth theory, the computational equation quantifies the interdependence of resistivity, frequency, and depth. The origin of high-frequency negative phases in impedance analysis—commonly attributed to inductive reactance effects or measurement interference—remains undetermined in current research.

### 4.2. Data Processing

The quality of the derived results is contingent upon both data reliability and the robustness of the processing workflow. The systematic processing protocol applied to the dataset comprises the following two critical stages:

(1) Outlier data removal: Anomalous data points were systematically excluded, primarily arising from topographic discontinuities (e.g., abrupt elevation changes at the cliff edges) or suboptimal electrode coupling during field measurements.
(2) Static effect mitigation: A pre-inversion correction was implemented to address the pronounced static effect induced by shallow subsurface conductive heterogeneities. Following the methodology proposed by Torres-Verdín and Bostick (1992) [15], low-pass filtering was applied using a Hanning window (Equation (1)) to suppress high-frequency noise components, thereby enhancing the signal-to-noise ratio of the processed data.

(1) $$h(x)=\left\{\begin{array}{ll} \frac{1}{W}(1+\cos\frac{2\pi x}{W}) & \left|x\right|\leq W/2\\ 0 & \left|x\right|>W/2 \end{array}\right.$$

In Equation (1), *x* represents the filter length (i.e., variable separation between measurement points) and *W* denotes the window span. The filter *h*(*x*) comprises multiple discrete points. For two distinct filter configurations, the 7-point filter exhibits a superior vertical artifact suppression compared to the 3-point configuration due to its enhanced spatial sampling, which has been successfully verified [13].

While topographic corrections are typically crucial for CSAMT data interpretation in mountainous terrain, this procedure was omitted in the current study given the survey area’s negligible elevation variations (<10 m relief).

**Figure 3:**
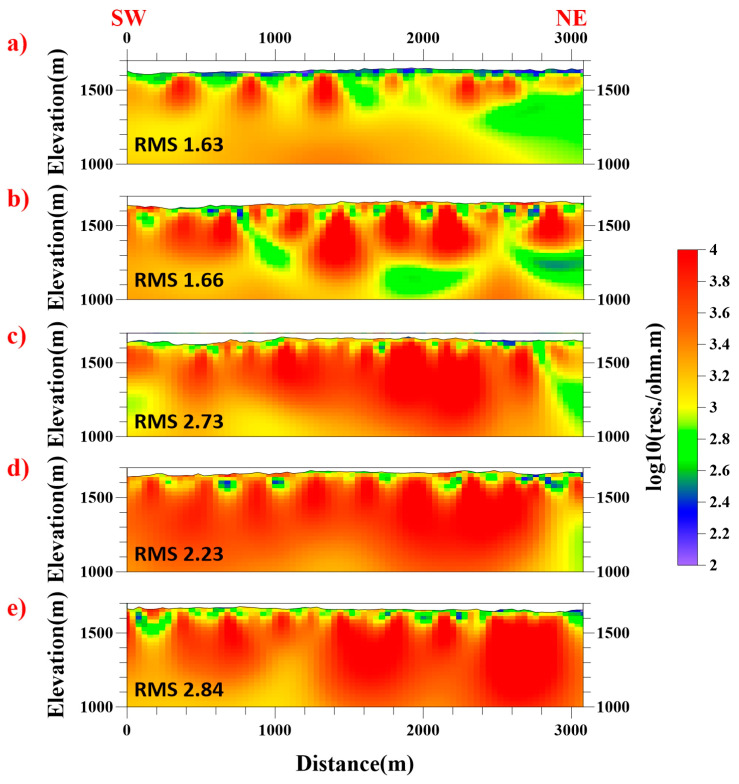
Two-dimensional resistivity models for five profiles. (**a**) N5; (**b**) N4; (**c**) N3; (**d**) N2; (**e**) N1.

The processing workflow concluded with apparent resistivity inversion, for which the cross-sectional data were partitioned into shallow and deep domains. Despite ongoing advancements in 3D CSAMT inversion methodologies, computational complexity and source characterization challenges currently limit their practical implementation. Consequently, we employed a 2D smooth-model inversion approach [16,17] exclusively on far-field data. This robust inversion framework transforms CSAMT observations into resistivity models through the iterative adjustment of model parameters, initialized using 1D inversion results. The algorithm combines finite element modeling with smoothness-constrained least-squares optimization to minimize discrepancies between calculated and observed apparent resistivity/impedance phase data. After eight iterations, the solution converged with a final RMS misfit of 1–3% (Figure 3). The geological interpretation of the inversion results was subsequently conducted through integration with regional stratigraphic constraints and borehole data.

## 5. Inverted Results and Interpretation

### 5.1. Sample Resistivity Measurement

The CSAMT method is a frequency-domain electromagnetic technique utilizing subsurface resistivity contrasts. This geophysical parameter is governed not only by lithology but also by structural features and fluid saturation levels. Prior laboratory analyses of resistivity properties for granitic lithologies from the Beishan study area have been systematically characterized (Table 1) [13]. The sample dimensions are 50 mm in length and 25 mm in diameter, with testing conducted in accordance with the SY/T 6712-2023 industry standard under ambient laboratory temperature conditions.

### 5.2. Interpretation Results

Profile N3 is situated centrally among the five NE-trending CSAMT profiles (see Figure 1) and passes near borehole BS32. Its geological structural section is considered representative; thus, it was selected for detailed interpretation.

**Figure 4:**
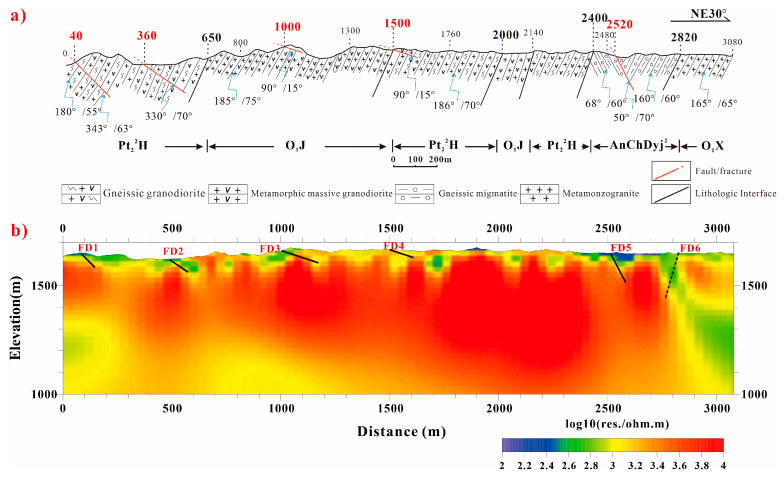
Geological section and interpretation of profile N3. (**a**) Field-measured geological section; (**b**) interpretation map.

Figure 4 presents the geological section and inversion interpretation results for Profile N3. Overall, the resistivity characteristics are relatively high, dominated by medium-to-high and high resistivity. Five distinct resistivity boundaries are observed horizontally along the profile, at approximately 40 m, 360 m, 1000 m, 1500 m, and 2520 m. These key boundaries correspond well with the geological features observed at the surface.

The first four boundaries (at 40 m, 360 m, 1000 m, and 1500 m) correspond to anomalies characterized by medium resistivity and exhibit a depth extent of less than one hundred meters. In contrast, the resistivity boundary at 2520 m is pronounced and extends to a significantly greater depth.

At 40 m: A fault (FD1), striking approximately 70°, dipping NW with a dip angle of about 63°, and having a fracture zone width of about 4 m, is developed within medium–fine-grained gneissic granodiorite (Pt_2_^2^H). Because this fault intersects profile N3 obliquely at an angle of 40° and dips NW, the fault at the 40 m point on the geological section corresponds to the resistivity boundary observed at the 80 m point on the inversion profile.

At 360 m: Fault FD2 is observed at the surface within medium–fine-grained gneissic granodiorite (Pt_2_^2^H). This fault has a width exceeding 2 m and contains intermediate-acidic dikes. It strikes 60°, dips NW, and has a dip angle of about 70°. Similarly, due to its oblique intersection angle of 30° with the survey line direction and its NW dip, this feature is laterally displaced to the 480 m point on the CSAMT profile.

At 1000 m and 1500 m: The geological section shows minor faults FD3 and FD4, respectively. These faults have very gentle dip angles (only 10–15°), dip eastward (90°), and exhibit narrow compression fracture zones approximately 0.5 m wide. Both are exposed near the surface. Similar minor faults are observed in several profiles within the northern Xinchang area, but their resistivity expression on CSAMT profiles is not distinctly visible. This lack of distinct expression is likely attributable to their very shallow dip angles and proximity to the surface. They are included in the geological section primarily due to their geological significance. These minor faults exhibit compressional characteristics, indicating the presence of an approximately E-W-oriented compressive stress field in this region during a specific geological period.

At 2520 m: Fault FD5, striking NW and dipping NE, is observed at the surface. Based on its strike extension and geomorphological expression, it is interpreted as the same fault traversing the northeastern segments of Profiles N5 and N4. Trenching reveals that the scale of this fault zone diminishes significantly as it extends into profile N3.

Near 2820 m: A resistivity boundary is located at a lithological contact, manifested as a relatively broad and deep medium-to-low resistivity band, inferred to be a fault (FD6). This feature is likely caused by the intrusion of a series of intermediate-acidic dikes along this fault zone and its margins. These intrusions introduced hydrothermal fluids, resulting in wall–rock alteration and the relative enrichment of metallic elements, thereby forming a relatively broad alteration zone. Consequently, this produces the wide and deep medium-to-low resistivity band observed on the CSAMT profile.

## 6. Discussion

### 6.1. Inversion Fit Comparison at Near-Borehole Points

**Figure 5:**
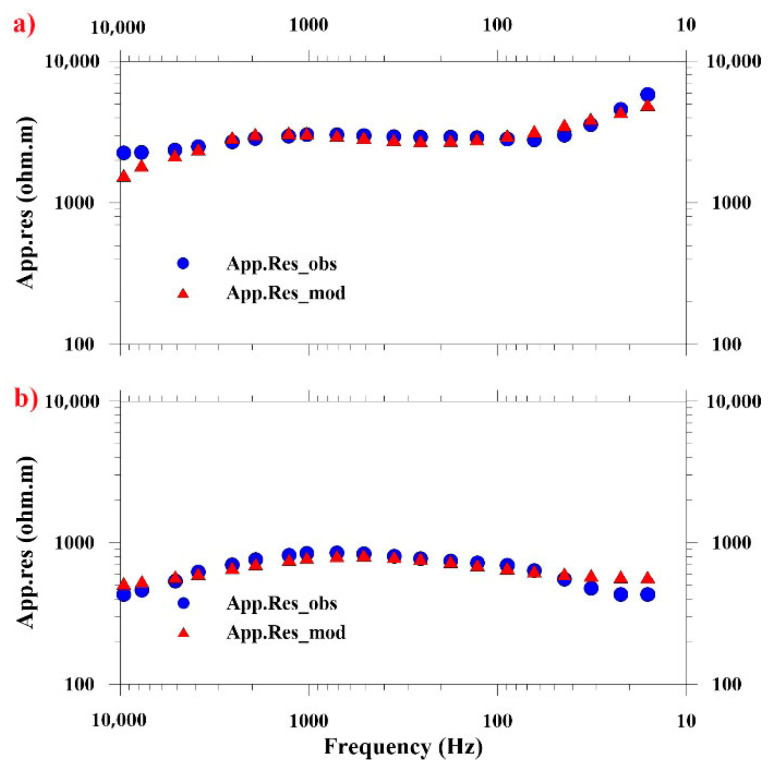
Modeled versus observed resistivity data at measurement sites adjacent to boreholes BS32 and BS33. (**a**) Station 1740 (Profile N2) near BS33; (**b**) Station 1520 (Profile N3) near BS32. The modeled resistivity curves (red triangles) closely match field observations (blue circles), demonstrating spatial variations in subsurface conductivity.

Anomalously low resistivity zones were identified within medium- to fine-grained gneissic granodiorite formations and fracture-controlled, partially saturated granitic units. The resistivity of the granodiorite matrix exhibited significant variability (1–2 orders of magnitude), correlating directly with mica content variations in the mineral assemblage. As shown in Figure 5, a notable congruence between field measurements and modeled responses was observed proximal to validation boreholes BS32 and BS33 (RMS error < 3), validating the inversion scheme’s reliability. Vertical deep boreholes BS32 and BS33 were planned for the exploration of geological conditions around the main shaft of the URL. The depths of these boreholes are about 600 m. The BS32 and BS33 boreholes are primarily composed of medium–fine-grained biotite granodiorite. The entire borehole exhibits highly intact rock conditions, with no observed fractured rock intervals [14]. The borehole cores were comprehensively logged in terms of lithology and structure and degrees of weathering and fracturing; the RQD (Rock Quality Designation) index was determined, showing very good rock integrity at the URL site [4].

### 6.2. Site Suitability Assessment for Underground Laboratory Construction

#### 6.2.1. High-Resistivity Zones as Indicators of Intact Rock Mass

**Figure 6:**
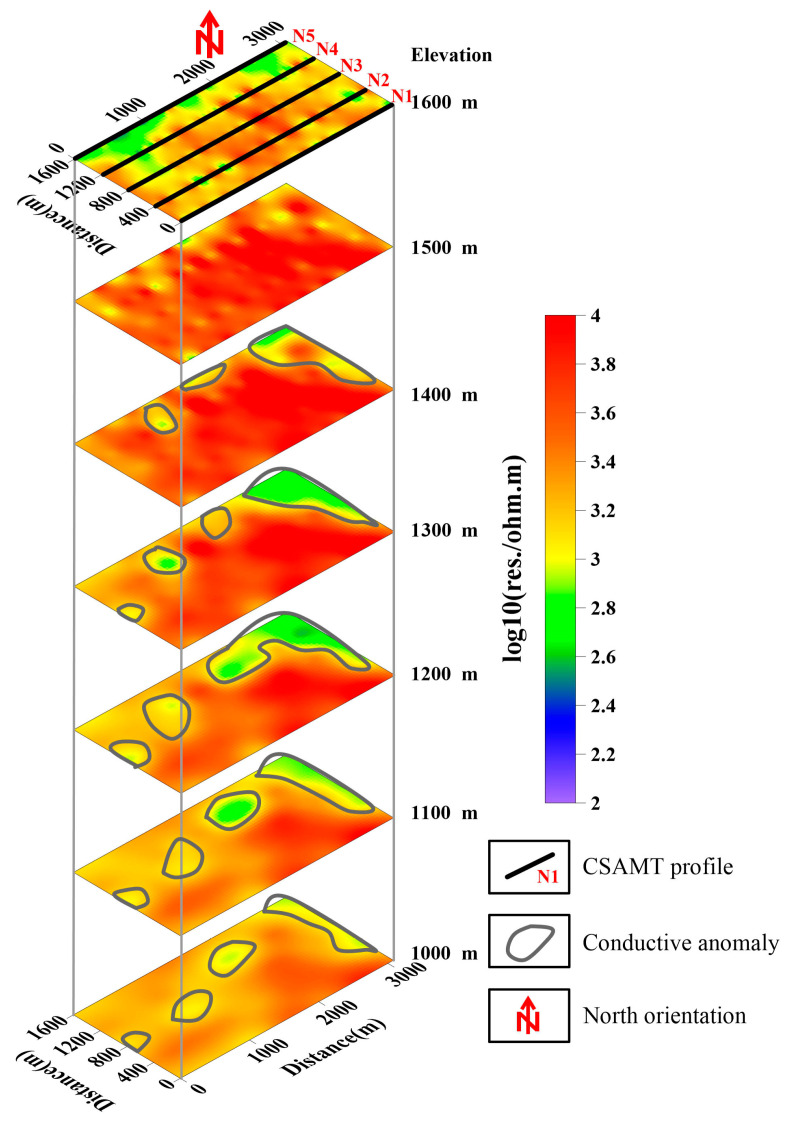
Resistivity slices at different depths.

The 2D inversion results (Figure 3) reveal predominantly elevated resistivity values (>1000 Ω·m) across the surveyed profiles. However, the distinct low-resistivity anomalies (200–500 Ω·m) identified along profiles N4 and N5 correlate with steeply dipping fractured zones, potentially indicating faults/structures. While these conductive features exhibit apparent lateral continuity in cross-section, integration with surface geology and borehole data constrains their vertical extent on a small scale. In contrast, profiles N1-N3 demonstrate relatively homogeneous resistivity structures dominated by high-resistivity bedrock (>2000 Ω·m) below a 200 m depth, with moderate anomalies (500–800 Ω·m) confined to shallow weathering layers. The resistivity depth slices in Figure 6 further delineate localized conductive anomalies in the northeastern and western sectors of the study area, contrasting with the electrically resistive background (>1500 Ω·m) prevalent elsewhere.

Intact rock exhibits a high resistivity due to minimal ionic conduction pathways, whereas fractures/clay fillings reduce resistivity via enhanced conductivity. High-resistivity anomalies (>3000 Ω·m) delineated through CSAMT effectively indicate zones of intact rock mass with minimal fracturing, as demonstrated by the drilling validation in granite areas [18]. A 92% accuracy in predicting rock mass integrity (model validation metric, *R*^2^ = 0.87) was confirmed by correlating ERT (Electrical Resistance Tomography) data with 35 borehole-measured *Kv* (Intactness Index of Rock Mass) values. High-resistivity anomalies (>1000 Ω·m) typically signify low-fracture-density rock mass, as validated by borehole-derived integrity coefficients (*Kv* > 0.55) [19].

#### 6.2.2. Geological Suitability of Xinchang Site for HLW Geological Disposal

Controlled-source audio-frequency magnetotelluric (CSAMT) surveys, integrated with field geological investigations, indicate favorable geological conditions at the Xinchang site for constructing a high-level radioactive waste geological repository. This conclusion is supported by the following evidence.

**Geomorphological Characteristics:**

The northern Xinchang area exhibits a distinctive geomorphology comprising a series of low-relief hills. These hills display minimal relative elevation differences, with their summits converging toward a common peneplanation surface. Intervening depressions are predominantly broad and flat, rarely forming narrow, deep gullies. Even larger gullies influenced by fault structures exhibit gentle gradients. Gully infill consists of thin sand–gravel deposits (typically centimeters to decimeters thick; rarely exceeding 1 m), with frequent bedrock exposures. This geomorphology indicates a negligible neotectonic activity since the Late Neogene, reflecting a regime of stable, slow uplift. Consequently, the region exhibits favorable crustal stability for repository development.

**Climatic Conditions:** The arid climate features minimal annual precipitation and underdeveloped groundwater systems. These conditions result in exceptionally weak chemical weathering. Weathering layers are thin, with surface and near-surface rocks retaining freshness—additional advantageous factors for repository construction.

**Joint Characteristics:** Rock masses are sparsely fractured by predominantly tight, closed shear joints. This discontinuity pattern contributes to overall high rock mass quality.

**Fault Characteristics:**

The Xinchang site is situated within a relatively stable block bounded by the F6 and F7 faults and secondary faults (F31, F32, and F34), exhibiting excellent rock mass continuity and suitability for deep engineering projects [14]. Faults within the pre-selected area exhibit the following key attributes:

① Sparsely distributed fractures preserve large volumes of intact rock mass within localized domains.

② Most fracture zones are narrow (typically several meters wide), minimizing rock mass damage.

③ Fractures often terminate within short along-strike distances. A fracture observed on one survey line frequently fails to be detected on adjacent lines, indicating discontinuous lateral propagation.

④ Downward penetration of fractures is restricted, with limited vertical extent inferred from subsurface data.

**Dominant Orientations and Mechanics:** NE and NW strikes predominate, with subordinate E-W trends. Most faults display compressional–shear kinematics, indicating tight closure, which is conducive to rock mass stability.

**Dike Emplacement and “Healing” Effects:** Mafic dikes (e.g., N-S- to NNE-trending diabase) commonly intrude tensile fractures. CSAMT profiles show no clear resistivity boundaries at these locations, with persistent medium–high resistivity values suggesting the dike-induced “healing” of discontinuities. Intermediate-felsic dikes also intrude fault zones. Some show minimal deformation, while others develop compressional foliation or lenses, confirming fault closure under compressional/transpressional stress. Such sealed faults pose significantly lower stability risks than extensional faults [17].

**Post-Emplacement Stability:** Felsic/granitic dikes intruding faults from multiple directions remain intact and undeformed. This indicates no fault reactivation since dike emplacement, further demonstrating long-term rock mass stability.

For decades, the Beijing Research Institute of Uranium Geology has conducted extensive geological investigations in the Beishan Pre-selected Area, Gansu Province, implementing targeted engineering excavations. These efforts have generated invaluable data for assessing rock mass integrity and stability. The resulting scientific achievements provide critical reference value and guidance for geophysical exploration programs. On 17 June 2021, the construction of China’s Beishan Underground Research Laboratory (URL) commenced [20]. In September 2024, China opened 12 nuclear research facilities—including the Beishan URL—to global partners [21].

## 7. Conclusions

We present a CSAMT investigation conducted at a pre-selected deep geological repository site in the arid Beishan region, northwestern China. To optimize signal transmission fidelity, we implemented saline-solution electrode coupling for both transmitter–receiver pairs. A modified Hanning-window spatial filter effectively mitigated static shift artifacts in the raw dataset. The two-dimensional inversion of coupled apparent resistivity and phase datasets enabled the reconstruction of subsurface conductivity structures to 650 m depth, revealing critical geological discontinuities. The inverted models demonstrate a correlation coefficient with independent constraints including borehole logging (BS32/BS33), trench stratigraphy, and surface structural mapping. Notably, the CSAMT-derived conductive anomalies (<300 Ω·m) align precisely with surface-exposed fault zones exhibiting intense cataclastic deformation, interpreted as pathways for Late Paleozoic acidic dike emplacement during intracontinental tectonic reactivation. Lithological analysis confirms the host medium comprises predominantly fresh biotite monzonitic granite (2500–4000 Ω·m) and minor granodiorite bodies—both exhibiting a low fracture density and favorable geomechanical properties for underground research laboratory (URL) development.

The results demonstrated a strong agreement between inverted models and borehole data, revealing a 600-m-thick competent granite layer (>2500 Ω·m) beneath surface weathering zones. This geological configuration, combined with the area’s crustal stability and minimal human activity, confirms the site’s compliance with IAEA repository safety standards (SSG-14) [22]. Based on the geophysical characterization results, we recommend the following integrated actions to bridge scientific findings with repository implementation: (1) optimize URL construction by siting critical infrastructure within identified high-resistivity zones (>2500 Ω·m) at depths of 300–800 m, employing TBM excavation techniques in fracture-prone sectors; (2) establish a 4D resistivity monitoring network leveraging baseline data to detect early containment risks, with Δ*ρ*/*ρ*_0_ > 15% serving as a hydro-mechanical instability threshold; and (3) initiate coupled modeling studies integrating the resistivity structure into TOUGH-FLAC simulations to quantify fracture-permeability evolution under thermal–hydrological–mechanical–chemical (THMC) stresses. This multidisciplinary approach—exemplified by ONKALO’s EDZ monitoring protocols—will transform geophysical anomalies into predictive safety performance metrics for China’s HLW program.

## Figures and Tables

**Table 1:** Average resistivity values of the main lithologies in the Beishan area, as recorded in rock sample tests.

Lithology	Average Resistivity (ohm·m)
Diabase	2197
Massive granodiorite	12,206
Fine-grain granite	9679
Diorite porphyrite	9860
Medium- to fine-grain gneissic granodiorite	2622
Medium- to fine-grain granodiorite	6957
Migmatite	5060

## Data Availability

The raw data supporting the conclusions of this article will be made available by the authors on request.
